# Real time US –guided ventricular catheterisation: a single centre retrospective audit

**DOI:** 10.1186/2045-8118-12-S1-P1

**Published:** 2015-09-18

**Authors:** Erjon Agushi, James Montgomery Barber, Konstantina Karabatsou

**Affiliations:** 1 Salford Royal NHS Foundation Trust, UK

## Introduction

Suboptimal shunt or external ventricular device (EVD) catheter placement can result in significant morbidity, requiring system revision and consequently prolonging in-hospital stay. Multiple incorrect trajectories can potentially cause further complications and neurological deficits. While experienced operators can achieve relatively high success rates with the craniometric technique, the failure rates can be even greater than 20%, in certain studies.

## Design

Retrospective audit of documented method of guidance technique and subsequent clinical/radiological outcomes.

## Objective

The objective of our study was to compare the clinical benefits of real-time Ultrasound guidance in enhancing ventricular catheter insertion versus the standard craniometric-guided free-hand technique.

## Methods

Operative records, clinical outcomes and postoperative scans of the patients who underwent EVD or shunt insertion from between March 2013 to March 2014 at a single centre were reviewed retrospectively.

## Results

A total of 162 patients' clinical notes and scans were reviewed. 22 were excluded due to unclear documentation, missing operative notes or postoperative scan having not been performed. 78 underwent an EVD insertion, mainly for secondary hydrocephalus due to SAH, whereas 62 had insertion of a VP shunt as a primary procedure. Use of US to visualize the ventricles was documented in 57.9% (56.4% in EVDs vs. 59.7% in shunts), while usage of real-time US guidance of catheter insertion was recorded in 42.1% (39.7% in EVDs vs. 45.2% in shunts).

Incorrect or suboptimal positioning was significantly higher in the free-hand cohort as compared to the US guided group (22.3% vs. 5.1% respectively, p=0.005). Only one shunt was revised due to dysfunction in the real-time US guided group vs. 3 shunts and 5 EVDs in the free-hand group (1.7% vs. 10%, p=0.049). Postoperative focal deficits were only recorded being present in patients undergoing free-hand insertion.

## Conclusions

Insertion of ventricular catheters under US guidance enables safe and optimal catheter placement, reducing significantly the rate of revisions and subsequent morbidity.

